# Recent advances in understanding and managing diverticulitis

**DOI:** 10.12688/f1000research.14299.1

**Published:** 2018-06-29

**Authors:** Carola Severi, Marilia Carabotti, Alessia Cicenia, Lucia Pallotta, Bruno Annibale

**Affiliations:** 1 Department of Internal Medicine and Medical Specialties, University Sapienza of Rome, Viale del Policlinico 155, 00161 Rome, Italy; 2 Medical-Surgical Department of Clinical Sciences and Translational Medicine, University Hospital S. Andrea, University Sapienza of Rome, Via di Grottarossa 1035-1039, 00189 Roma, Italy

**Keywords:** Diverticulitis, Risk factors, Prevention, Therapy, Diet, Microbiota, Colonic muscle, Drugs

## Abstract

In the past few decades, the increasing socioeconomic burden of acute diverticulitis (AD) has become evident, and with the growth of the population age, this significant economic impact will likely continue to rise. Furthermore, recent evidence showed an increased rate of hospital admissions especially evident among women and younger individuals. The natural history and pathophysiology of this clinical condition is still to be fully defined, and efforts continue to be made in the identification of risk factors and the establishment of relative preventive strategies. The actual therapeutic strategies aimed to modulate gut microbiota, such as rifaximin or probiotics, or to reduce mucosal inflammation, such as mesalazine, present a relatively poor efficacy for both the prevention of the first AD episode (primary prevention) and its recurrence (secondary prevention). In the last few years, the main goal achieved has been in the management of AD in that uncomplicated AD can, to a larger extent, be managed in an outpatient setting with no or little supportive therapy, a strategy that will certainly impact on the health costs of this disease. The problem of AD recurrence remains a topic of debate.

The aim of this review is to present updated evidence on AD epidemiology and relative open clinical questions and to analyze in detail predisposing and protective factors with an attempt to integrate their possible modes of action into the several pathogenic mechanisms that have been suggested to contribute to this multifactorial disease. A unifying hypothesis dealing with the colonic luminal and extra-luminal microenvironments separately is provided. Finally, evidence-based changes in therapeutic management will be summarized. Because of an ascertained multifactorial pathogenesis of uncomplicated and complicated AD, it is probable that a single ‘causa prima’ will not be identifiable, and a better stratification of patients could allow one to pursue tailored therapeutic algorithm strategies.

## Introduction

Colonic diverticula, sac-like herniations of the colonic mucosa and submucosa through the muscle layers, represent a common gastrointestinal condition in the Western world with a prevalence that grows with age, from less than 10% in people younger than 40 years of age to 50–66% after 80 years of age ^1, 2^. While most people with colonic diverticula remain asymptomatic, making diverticulosis not a disease per se, around 20% of patients will develop diverticular disease (DD) when they experience abdominal symptoms (i.e. symptomatic uncomplicated DD [SUDD]) and 1–4% will develop acute diverticulitis (AD) ^3^. The natural history and pathophysiology of this clinical condition are still under definition. Clinical scenarios of DD and its natural history have been recently summarized ^4^. In the past few decades, the increasing socioeconomic burden of DD has become evident, and efforts have been made to identify risk factors to establish relative preventive strategies and to achieve more standardized treatment approaches.

The aim of this review is to present updated evidence on AD epidemiology and relative open clinical questions and to analyze in detail predisposing and protective factors with the attempt to integrate their possible modes of action into the several pathogenic mechanisms that have been suggested to contribute to this multifactorial disease. Finally, evidence-based changes in therapeutic management will be summarized.

## The clinical problem and its impact on health costs

AD is an inflammatory process that involves one or more colonic diverticula, often associated with pericolonic inflammation that is classified as uncomplicated or complicated, the latter being characterized by the presence of abscesses, fistulas, peritonitis, and colonic stenosis. Complications most commonly occur with the first episode of AD ^5^. AD clinical classification is mainly based on the use of modified Hinchey's criteria derived from contrast-enhanced computerized tomography (CT) imaging ^6^ or on the more recent German guidelines ^7^. However, an important role in AD diagnosis is also covered by abdominal ultrasound (US), which, in the hands of experienced investigators, can be used as a sensitive and specific diagnostic technique, limiting the use of CT after negative or inconclusive abdominal US ^7– 10^.

Recent epidemiological studies have confirmed the increase of hospital admissions for AD in recent years ^11^. A previous epidemiological analysis, carried out in the USA in 2012 ^12^, showed that diverticulitis was the third most-common gastrointestinal diagnosis from hospital admission and the leading indication for elective colon resection, with an increase of 41% from 2000 and an estimated cost of 2.6 billion dollars per year. A more recent analysis showed that the national cost of diverticulitis-related emergency department visits in the USA, from 2006 to 2013, increased by 105% ^13^. Similarly, a recent observational analysis based on real-world data from an Italian region reported that direct healthcare costs for episodes of diverticulitis from 2008–2014 amounted to approximately 11.4 million euros, of which 95.5% was for hospitalizations ^14^. With the growth of the population age, this significant economic burden is likely to continue to rise.

Currently, the increased rate of hospital admissions is especially evident among women and younger individuals ^5, 15^. Gender differences are age related in that hospital admissions for AD are predominant in men among subjects younger than 45 years of age, the opposite being true at older ages ^11^. Besides, even if older patients display the highest rate of hospital admissions for AD, the increased rate of hospitalization is entirely accounted for by the younger cohorts of patients ^13^.

## Open clinical questions

Recent randomized controlled trials (RCTs) confirmed, and national guidelines recommended, that patients with uncomplicated AD are eligible for outpatient treatment without the use of systemic antibiotics, which should be used in complicated patients instead ^16– 19^. This conservative management strategy, if adopted, could positively influence the relative economic burden on health costs. However, the problem of AD recurrence remains a topic of debate.

In a recent retrospective population-based cohort study of 65,162 patients identified with a first episode of AD, the rate of hospital admission for recurrence was 11.2% ^20^, lower than was previously reported ^21^. This recurrence rate was greater in younger people and women ^5^. Generally, the first episode is the most severe, with only 2% of recurrences resulting in a complicated case. Surgical treatment does not prevent the risk of recurrence, the rate of which is around 15% ^22, 23^.

Also, AD seems to predispose patients to developing long-term chronic nonspecific abdominal symptoms, similar to those observed in post-infectious irritable bowel syndrome ^24^, with a higher prevalence after severe AD ^25^. The proportion of patients that develop chronic abdominal pain after AD seems to be influenced by the type of treatment of the first episode, with a lower prevalence after elective laparoscopy (11%) compared to conservative treatment (39%) ^26^.

As for now, the underlying mechanisms and risk factors that contribute to AD and its recurrence still need to be clarified. The actual therapeutic strategies aimed to modulate gut microbiota, such as rifaximin or probiotics, or to reduce mucosal inflammation, such as mesalazine, present a relatively poor efficacy for the prevention of the first AD episode (primary prevention) and its recurrence (secondary prevention) (see later). A definite assessment of AD-predisposing or -protective factors and these relative mechanisms could greatly contribute to patients receiving the best strategy for prevention, further reducing health costs, and improving the management of DD.

## Risk factors for acute diverticulitis and relative recurrence

The identification of risk factors for AD has been the scope of several studies for the past few decades. In the last few years, the main concern has been to clarify whether or not factors associated with the first episode are also involved in AD recurrence ^27^. Predisposing and protective factors for AD are summarized in Table 1. Among anthropometric features, obesity has been confirmed to be a risk factor for AD by a population-based cohort study ^28^ and a recent systematic review and meta-analysis of prospective studies ^29^, a risk previously reported in men in whom waist-to-hip ratio was also associated with the risk of diverticular complications ^30^. As far as smoking is concerned, a lifestyle risk factor already known to be associated with AD, recent evidence showed that it further represents an increased risk of developing complicated AD ^31, 32^ and requiring surgery ^33^. Red meat intake, particularly of unprocessed meat, was identified as the main dietary risk factor for diverticulitis in a prospective Health Professionals Follow-Up Study (1986–2012) of 46,461 men ^34^.

**Table 1.** Summary of predisposing and protective factors associated with primary and secondary prevention of acute diverticulitis.

Predisposing	Protective	Need to be confirmed	Irrelevant
*A* *nthropometric and anatomic features* - BMI - waist circumference - waist–hip ratio - pancolic diverticula *L* *ifestyle* - smoking *D* *iet* - red meat intake *D* *rugs* - aspirin - NSAIDs - corticosteroids - opioids	*L* *ifestyle* - vigorous physical activity *D* *iet* - high-fiber intake *D* *rugs* - statins *O* *thers* - increased vitamin D levels	- alcohol - younger age - female gender >50 years old - genetic factors • family history • *TFNSF15* polymorphisms • *LAMB4* variants - calcium antagonists - comorbidities: • cardiovascular diseases • chronic obstructive pulmonary disease • end-stage renal disease	*D* *iet* - nuts - popcorn - corn - fine or coarse grains - coffee

BMI, body mass index; NSAIDs, non-steroidal anti-inflammatory drugs

A systematic review and meta-analysis ^35^ confirmed that non-steroidal anti-inflammatory drugs (NSAIDs), which are already known to be risk factors for AD ^36^ and perforation ^37^, as well as steroids and opioids, presented an increased odds ratio for perforation and abscess formation.

Among protective factors, vigorous physical activity, as was previously reported ^38^, was confirmed by a recent meta-analysis to be inversely related to diverticulitis ^29^ but not by a population-based cohort study ^28^. As far as diet is concerned, no recent further evidence is available on the lower risk of hospitalization for AD with a vegetarian diet and a high intake of dietary fibers ^39, 40^. Instead, statins, which have already been associated with a reduced risk of perforation ^35, 37^, have also been recently reported to be associated with a reduced risk of emergency surgery ^41^. Finally, vitamin D serum levels, which when high pre-diagnostically have been associated with a low risk of AD among patients with diverticulosis ^42^, have been related to the severity of DD endoscopic appearance ^43^.

Some known risk factors still need to be confirmed. Evidence regarding alcohol consumption and DD are discordant, with some studies showing a positive association ^44, 45^ and others not ^46^. Regarding drugs, the potential protective effect of calcium channel blockers in reducing the rate of diverticular complications observed in case-control analyses ^37, 47^ needs to be confirmed. Also, the recent epidemiological evidence that reports a higher prevalence of AD among women older than 50 years of age ^11, 48^, younger individuals ^5, 11, 13^, and patients with end-stage renal disease ^49^, as well as the association of cardiovascular disease and chronic obstructive pulmonary disease with complicated DD ^28, 50^, needs to be confirmed and explained.

Finally, recent studies corroborate the previous observation of a strong familial aggregation both in diverticulosis and in diverticular complications, suggesting a role for genetic factors ^51^. A relationship has been reported between complicated diverticulitis and a single nuclear polymorphism in the *TFNSF15* gene, a T-cell maturation receptor gene associated with other colonic inflammatory diseases ^52^, and between early onset diverticulitis or unrelated sporadic diverticulitis and variants in the laminin β4 gene ( *LAMB4*), which codes for constituents of the extracellular matrix that regulate the function of the enteric nervous system ^53^.

Of note, even if clinicians frequently advised patients to avoid some foods, such as nuts, corn, and popcorn, these are not associated with an increased risk of diverticulitis ^54^, and the same is true for fine or coarse grains ^28^. Regarding the possible role of coffee, a previous study did not report any association between coffee use and DD ^44^, similar to a recent cross-sectional study that did not observe any significant difference between coffee drinking and diverticulosis compared to SUDD or previous diverticulitis ^55^.

An assessment of risk factors associated with recurrent AD is on its way, even if more studies are required to better target secondary prevention. Currently, increased risks of recurrent AD, obtained with a logistic regression model, have been shown to be younger age, female sex, smoking, obesity, a Charlson comorbidity index score of more than 20, dyslipidemia, and first complicated AD ^20^. Other risks of recurrence are primary diverticulitis with abscess formation, an inflamed segment of more than 5 cm, multiple diverticula, and diverticula throughout the colon, with the risk of subsequent diverticulitis more than doubled after two earlier episodes of diverticulitis that further increases for every episode of recurrence ^56^.

## Pathophysiology and possible underlying biological mechanisms

**Figure 1.**
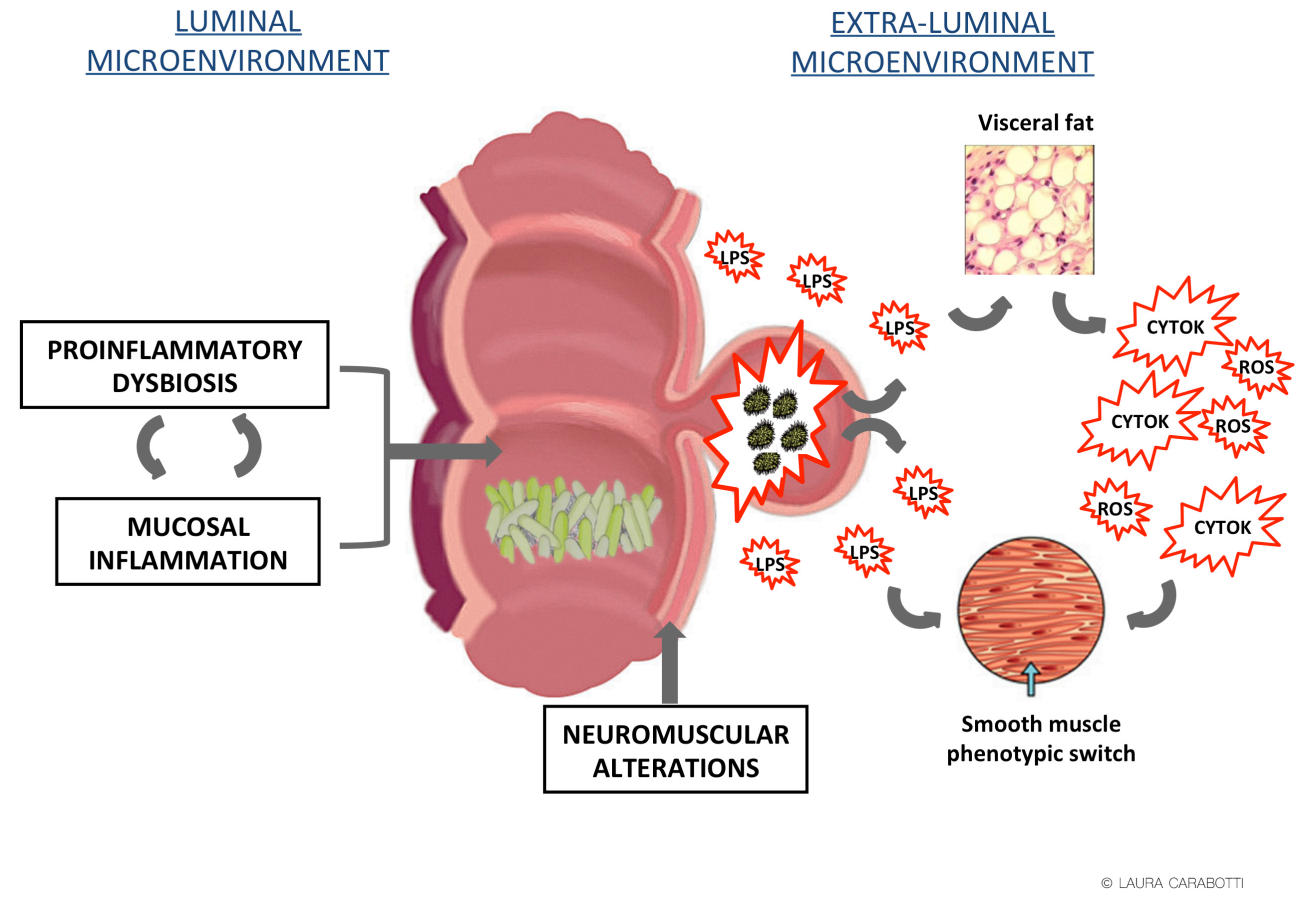
Pathogenic unifying hypothesis. CYT, cytokines; LPS, lipopolysaccharide; ROS, reactive oxygen species.

A unifying hypothesis to integrate the diverse risk factors and their contribution to the pathophysiology of AD has not yet been put forward. The current hypothesis asserts that the susceptibility of diverticula to inflammation is explained by local ischemia, retained stool, stercoral mucosal trauma by fecaliths, and diverticular wall distension that facilitates microperforations and favors bacterial translocation ^57^. Inflammation and infections can spread transmurally (peridiverticulitis), ending in different types of AD complications. The improvement of the clinical outcome of the disease obtained with antibiotics supports an involvement of bacteria in most AD complications. A dual inflammatory–infective contribution might then be considered in AD pathogenesis ^58, 59^ with likely different interconnections if considering the luminal colonic and the extra-luminal microenvironments ( Figure 1).

The luminal microenvironment is dominated by microbiota that has been suggested to play an important role in the pathogenesis of the disease ^60^. In uncomplicated DD, recent evidence indicates the presence of dysbiosis whose principal feature is the depletion of bacterial species with anti-inflammatory activity ^61^ that likely favors mucosal inflammation. Recently, in rodent models, it has also been reported that aging-associated microbiota promotes inflammation and intestinal permeability and increases pro-inflammatory cytokines in the blood ^62^, an aspect that could be relevant in DD, whose prevalence increases with age. Diet has an essential role in the maintenance of a healthy microbiota, and several types of food have been reported to be risk factors for AD likely through changes in microbiota composition. Among them, unprocessed red meat affects cecal microbial composition and metabolites ^63^ and aggravates experimental-induced colitis, an effect counteracted by the co-consumption of resistant starch, which is considered a type of fiber that provides the benefits of both insoluble and soluble fibers ^64^, an observation that could fit with the protective effects of a high-fiber intake in AD prevention. Fiber is known to possess anti-inflammatory effects and to have a eubiotic impact on gut microbiota ^65, 66^.

The presence of mucosal inflammation is still a matter of debate, except for DD-associated segmental colitis, a very rare mucosal inflammation sparing the rectum ^67^. Evidence derived from studies carried out on small numbers of patients supports its presence. A strong positive correlation has been reported between activated CD68 ^+^/CD163 ^+^ macrophages and complicated sigmoid diverticulitis ^68^, and an increase in the mast cell population has been found in the mucosal peridiverticular region ^61^. iNOS and NO release, which are expressed in an inflammatory context, are increased in colonic mucosa, presenting a progressive trend from diverticulosis to SUDD with previous diverticulitis ^69^. Similarly, higher chronic inflammatory infiltrates and inflammatory cytokine expression have been found in the affected tissue in patients after severe versus non-severe diverticulitis ^25^. However, the presence of mucosal inflammation has not been confirmed by a recent study carried out in a large number (255) of patients, at least neither in diverticulosis nor in SUDD ^70^. The absence of clear evidence regarding the efficacy of mesalazine in preventing both AD and its recurrence fits with these discrepancies.

The confirmation of an inflammatory mucosal context has several implications. Firstly, inflamed microenvironment in the gut drives bacterial dysbiosis ^71^, favoring a vicious cycle between dysbiosis-driven inflammation and inflammation-driven dysbiosis. Secondly, mucosal inflammation and dysbiosis might lead to dysmotility ^72^, another pathogenic factor associated with DD.

Dysmotility is present in both uncomplicated and complicated DD owing to a summation of different abnormalities ^73^. Increased motility index and abnormal propulsive activity have been reported in patients with DD ^74^, and enhanced contractile response has been observed in DD muscle strips, with upregulation of substance P and muscarinic M _3_ receptors ^75– 77^. In addition, specific abnormalities in longitudinal muscle relaxation and contents of neural nitric oxide and elastin have been reported ^78^, confirming old observations of elastosis confined to the longitudinal muscle layer with subsequent shortening of the teniae coli ^79^. A remodeling of the neuromuscular apparatus occurs in complicated DD, consisting of a muscular hypertrophy and architecture disarrangement with reduced content in density of myocytes and contractile myofilaments ^80^, an enteric neuropathy with hypoganglionosis, an imbalance in neurotransmitters, a deficiency of glial neurotrophic factors coupled to a nerve fiber remodeling ^81– 83^, and an increase in collagen deposition in the colon wall ^84^. An increase in collagen expression has been observed in human colonic circular and longitudinal smooth muscle cells from complicated DD, parallel to a switch from a contractile to a synthetic phenotype ^85^. Of note is also that young patients with AD present an altered ratio between collagen subtypes with respect to female and older patients ^86^.

As a whole, these alterations increase the rigidity and thickening of the colonic wall and subsequent loss of tensile strength that could squeeze the diverticulum orifice, favoring ischemia and stasis. The presence of a hypercontractile status may offer an explanation for the protective role reported for calcium channel blockers and conversely the increased risk of AD associated with opioids and NSAIDs. Opioids inhibit propulsive motility patterns ^87^, while NSAIDs, by inhibition of COX enzymes, reduce prostaglandin-driven vasodilatation and muscle relaxation ^88^. Of note, however, is that most of the available data have been obtained from surgical specimens of chronic stages of DD, and then it remains to be established whether neuromuscular alterations correspond to a primary or secondary post-inflammatory event. If the enteric neuromuscular pathology reflects a primary event, this could lead to disturbed intestinal motility patterns, increased intraluminal pressure, and ultimately to the formation of diverticula and relative inflammation, whereas if inflammatory events are considered as the driving force for the neuromuscular abnormalities, these changes would arise as secondary lesions resembling an associated pathology.

The scarce efficacy of the actual medical treatments aimed to counteract luminal pathogenetic factors, however, suggests that some other extra-luminal factors likely contribute to DD complex pathogenesis. Up to now, minor attention has been given to the alterations of the extra-luminal microenvironment. Microbiota-driven mucosal inflammation can favor the translocation of luminal bacteria from the diverticula wall to the perivisceral area with potential activation of receptors of the innate immune system, Toll-like receptors (TLRs), that can drive an inflammatory response in the surrounding tissues. In Crohn’s disease, the creeping fat surrounding the affected area was found to contribute to the overall inflammatory response ^89^ and to be associated with a structuring/fistulizing course of disease ^90^. Similarly, surrounding visceral fat has been hypothesized to play a pathogenic role in complicated DD ^91^. In this context, the clinically significant link that has recently been reported between visceral fat and the severity of the presentation of diverticulitis is of interest, with visceral to subcutaneous ratio likely representing a predictive value of more complicated disease ^92^, in accordance with obesity as a risk factor for AD.

Bacterial translocation from the diverticula wall to the perivisceral area can also activate TLRs expressed on human colonic muscular cells ^93^. LPS binding to TLR4 is capable of triggering persistent and long-term oxidation-driven phenotypic myogenic cellular alterations ^94^, which are conceivably associated with a smooth muscle cell ‘shift’ toward the synthetic phenotype ^95^, resulting in a functional impairment of human colonic smooth muscle cells. Massive intramuscular fibrosis of both muscle layers has been described in DD ^96^ and recently fibrosis, not predictable on endoscopic mucosal biopsies, has also been detected in DD submucosa ^97^. Of note is that maximum colonic wall thickness is one of the factors used to predict recurrent diverticulitis ^98^. This observed fibrotic trend could be influenced by NSAIDs, a known risk factor for AD and recurrences, that cause an enteropathy characterized by multiple short-segment strictures ^99^. Part of the NSAID-induced damage in the gastrointestinal tract is caused by an uncoupling of mitochondrial oxidative phosphorylation that favors oxidative stress ^100^. Likewise, the protective effect of statins could be related to their possible anti-fibrotic effects ^101^. The involvement of peridiverticular tissues might then contribute to the decrease in compliance leading to stiffer tissue that is more susceptible to tears, especially under conditions of increased luminal pressures favored by muscular hypertrophy.

## Acute diverticulitis management

AD clinical classification and risk stratification is based on CT, which is able to offer a more comprehensive evaluation of uncomplicated and complicated forms, and severity and management are graded with the use of modified Hinchey's criteria ^6^. More recently, a detailed classification of AD was proposed by German guidelines based on CT and clinical laboratory criteria ^7^ ( Table 2). Among AD, roughly two-thirds of patients present with uncomplicated diverticulitis ^102^.

**Table 2.** Acute diverticulitis management and classification according to modified Hinchey and German Society Gastroenterology classifications.

Modified Hinchey Classification	German society Gastroenterology Classification	Management
**1a** Confined pericolonic phlegmon and associated inflammation without an organized fluid collection	**1a** No peridiverticulitis **1b** Pericolonic phlegmon	Consider outpatients management Not routine use of systemic antibiotic *
**1b** Pericolonic abscess less than 4 cm in size, adjacent to the area of diverticulitis	**2a** Concealed perforation abscess ≤1cm	Hospitalization Bowel rest, parenteral fluids Systemic antibiotic
**2** Pelvic or inter-loop abscess, or abscess larger than 4 cm	**2b** Paracolic or mesocolic abscess >1cm	Hospitalization Bowel rest, parenteral fluids Systemic antibiotic Consider Ultrasonography- or CT-guided drainage Consider surgery
	**2c** Free perforation	Hospitalization Bowel rest, parenteral fluids, Systemic antibiotic Consider laparoscopic lavage/surgery
**3** Purulent peritonitis	**2c1** Purulent peritonitis	
**4** Fecal peritonitis	**2c2** Fecal peritonitis	

*except for immunocompromised patients and severe comorbidities

In this setting, an important role might be provided by abdominal US, which, in the hands of experienced physicians, can be used as a sensitive and specific diagnostic tool ^7– 10^. A multicenter study evaluating the accuracy of US compared with CT in unselected patients referred for acute abdominal pain to the emergency department showed that CT has a higher sensitivity compared to US in detecting AD (81% versus 61%; *p*=0.048) ^103^. Currently, however, a strategy for providing CT after negative or inconclusive US has been proposed ^7, 9, 10^.

There has been considerable focus over the past few decades on a conservative treatment strategy based on the administration of systemic antibiotics as well as on surgery either electively or in an emergency setting.

More than five years ago, Chabok *et al*. published the first prospective RCT in this area, the AVOD trial, showing that antibiotic treatment in patients with uncomplicated diverticulitis (without CT signs of abscess, fistula, or free air) neither accelerates recovery nor prevents complication or recurrences ^104^. The most recent multicenter RCT of observational versus systemic antibiotic treatment (DIABOLO trial: amoxicillin plus clavulanic acid 1.2 g four times daily intravenously for at least 48 hours, after which the route was switched to oral administration of 625 mg three times daily) for a first episode of CT-proven uncomplicated AD (Hinchey stages 1a and 1b) showed that observational treatment without antibiotics did not prolong recovery and can be considered appropriate in patients with uncomplicated diverticulitis ^19^. However, even if no significant differences between Hinchey stages 1a and 1b diverticulitis were found, it should be noted that the vast majority of patients included had a diagnosis of Hinchey stage 1a AD (90.1% in the observational and 94% in the antibiotic-treated group) with only a small percentage of patients with Hinchey stage 1b diverticulitis. Although these results suggested that antibiotics may not be necessary in patients with Hinchey stage 1b diverticulitis, currently no strong evidence to treat these patients without antibiotics is available, and more data need to be collected. In fact, a recent systematic review of national and international guidelines recommended treating small abscesses with antibiotics ^18^. Moreover, only short-term results in omitting antibiotics were reported by both cited RCTs (AVOD and DIABOLO trials). More recently, the long-term effects of omitting antibiotics in uncomplicated AD were assessed after 24 months’ follow-up of the DIABOLO trial ^105^. In the observational group, even if no significant differences were found in terms of recurrent diverticulitis and sigmoid resection, a higher number of elective resections was reported. Accordingly, the most recent European ^7– 10, 106, 107^ and American ^108^ guidelines suggest the non-routine use of systemic antibiotics in patients with uncomplicated AD ( Table 2). The need of hospitalization has been reconsidered as well, and a recent systematic review supported the safety, efficacy, and economic efficiency of an outpatient-based treatment approach ^109^.

The management of complicated AD depends on its severity and complexity, requiring hospitalization, bowel rest, parenteral fluids, and, in selected cases, surgery. Antibiotic therapy is part of the management of complicated diverticulitis, and recent European guidelines ^7– 10, 106, 107^ are in accordance in recommending the use of broad-spectrum antibiotics ( Table 2).

How to best treat complicated AD with surgery has been under debate and subject to notable changes recently. In particular, the number of episodes is not the only indication for surgery in patients with recurring diverticulitis: the individual case and course is also taken into account. A recent open-label randomized multicenter trial (DIRECT trial) randomized 109 patients after an episode of AD to receive surgical treatment or conservative management ^110^. After a brief follow-up of 6 months, elective sigmoidectomy resulted in a better quality of life (assessed by many specific questionnaires) compared to conservative management. These results, even if innovative, might be affected by the heterogeneity of patients included (both patients with recurrent diverticulitis and patients with persistent abdominal complaints). In this setting, clinicians should carefully assess the relationship between symptoms and colonic diverticula differentiating abdominal complaints from irritable bowel syndrome, whose prevalence might increase after an episode of AD ^24^.

Currently, the decision to perform an elective resection after one or more episodes of AD should be undertaken on a case-by-case basis, taking into account risk factors, complications, age, and severity of episodes as well as the patient’s personal circumstances and comorbidities (i.e. immunosuppressed patients) ^111, 112^.

Actually, the possible recognition of clinical or biochemical parameters that could be used to identify patients who progress from uncomplicated to complicated AD and to monitor the potential development of complications requiring immediate surgical intervention are matters of debate. Procalcitonin ^113^ and neutrophil count and white cell to lymphocyte ratio ^114^ have been proposed as accurate markers to differentiate complicated from uncomplicated diverticulitis, and higher levels of calprotectin, an inflammatory colonic mucosal marker, tend to be associated with more severe AD ^25^. Clinical predictors of early recurrences up to 6 months after a first episode of AD appear to be high C-reactive protein (CRP) levels ^115^, the presence of systemic inflammatory response syndrome, high pain score, and regular steroid or immunomodulator use ^116^. For now, the proper timing of surgically treating AD remains undetermined, with surgical resection probably being reserved for patients with severe recurrences. Abscess formation should be treated by ultrasonography- or CT guided drainage while patients with signs of free perforation should undergo immediate surgery. Because of advancements in interventional technologies and laparoscopic treatment methods, surgical therapy is primarily aimed towards the control of emergency situations and avoidance of Hartmann’s procedures ^112^.

## Medical strategies for acute diverticulitis prevention

Treatment protocols in DD, especially for AD prevention, have been recently summarized ^117^. In AD prevention, epidemiological studies suggested that people consuming the highest quantity of fiber had a 41% lower risk of developing DD (0.59, 0.46 to 0.78; *p*<0.001) in comparison with those consuming less fiber ^46^, with the reduced risk being strongest for cereal and fruit fiber ^39^. This is in accordance with many national guidelines ^7– 10, 106, 107^ supporting their use.

Regarding rifaximin, recent evidence ^118^ confirms that short monthly cycles of rifaximin with fiber supplementation may reduce the risk of AD occurrence, even if the number needed to treat (NNT) to prevent an episode of AD in one year was 57 ^119^. Despite the high NNT, Polish ^107^ and Italian ^9, 10^ guidelines recommend the use of rifaximin associated with fiber intake. Data regarding the use of rifaximin for the secondary prevention of AD are weak, and recent guidelines did not agree with each other, with some for ^8– 10^ and others against ^7, 108^ its use. The most recent open RCT evaluated the efficacy of one-year intermittent rifaximin plus fiber to prevent AD recurrence ^120^. After randomization, the underpowered number of patients included caused a study switch from evidence gathering to proof of concept. However, the authors reported that rifaximin was more effective compared to fiber alone in the secondary prevention of AD, with recurrence occurring in 10.4% versus 19.3% of patients, respectively ( *p*=0.033).

Data regarding the use of mesalazine are inconclusive and more studies are needed, even if some positive results emerged for AD primary prevention ^121^. Recently, the role of mesalazine in the secondary prevention of AD has been investigated by several RCTs ^122, 123^ that showed that mesalazine is not significantly superior to placebo in preventing AD recurrence. A recent Cochrane systematic review ^124^ confirmed the uncertain role for mesalazine and its effects on AD recurrence, and a similar conclusion was drawn by a recent meta-analysis ^125^.

## Conclusion: possible tailored therapeutic strategies

In the last few years, the main goal that has been achieved in the management of AD is that uncomplicated AD can, to a larger extent, be managed on an outpatient basis with no or little supportive therapy, a strategy that will certainly impact on the health-related costs of this disease. A second achievement, one that needs more controlled studies, is the finding that one primary preventive intervention in AD is diet, specifically an adequate intake of fiber. The preferred type of fiber still needs to be elucidated considering that a high content in a FODMAP diet could cause an increase in colonic gas and fluids due to fermentation ^126^.

Some other topics, mainly concerning AD recurrences, remain topics of debate. There is also a need of robust well-designed placebo-controlled RCTs that take into account the clinical history of the patient in order to achieve clearer evidence.

Because of an ascertained multifactorial pathogenesis of uncomplicated and complicated AD, it is likely that a single ‘causa prima’ will be not identifiable. In turn, it would be useful to stratify patients in order to separate those who could respond to lifestyle modifications from those with more aggressive disease who could be treated better with surgery. It is possible that besides age-dependent alterations, different pathogenic phenotypes might characterize more aggressive and complicated DD, such as those observed in younger people. A better stratification or a longer and more careful observation of the natural history of the diverticulosis versus diverticulitis process could be helpful to verify this hypothesis. It must be kept in mind that the actual therapeutic strategies (anti-inflammatory drugs, non-absorbable antibiotics, and probiotics), targeted towards luminal alterations, more easily demonstrated by the use of endoscopic samples, showed unsatisfactory efficacy in primary and secondary prevention. A better understanding of the different possible factors involved will be of great help in the planning of different possible therapeutic strategies, such as the use of nutraceuticals, and in pursuing tailored therapeutic algorithm strategies.

## References

[1] Stollman N Raskin JB: Diverticular disease of the colon. *Lancet.* 2004;363(9409):631–9. 10.1016/S0140-6736(04)15597-9 14987890

[2] Feuerstein JD Falchuk KR: Diverticulosis and Diverticulitis. *Mayo Clin Proc.* 2016;91(8):1094–104. 10.1016/j.mayocp.2016.03.012 27156370

[3] Shahedi K Fuller G Bolus R: Long-term risk of acute diverticulitis among patients with incidental diverticulosis found during colonoscopy. *Clin Gastroenterol Hepatol.* 2013;11(12):1609–13. 10.1016/j.cgh.2013.06.020 23856358 PMC5731451

[4] Cuomo R Barbara G Annibale B: Rifaximin and diverticular disease: Position paper of the Italian Society of Gastroenterology (SIGE). *Dig Liver Dis.* 2017;49(6):595–603. 10.1016/j.dld.2017.01.164 28215517

[5] Bharucha AE Parthasarathy G Ditah I: Temporal Trends in the Incidence and Natural History of Diverticulitis: A Population-Based Study. *Am J Gastroenterol.* 2015;110(11):1589–96. 10.1038/ajg.2015.302 26416187 PMC4676761

[6] Kaiser AM Jiang JK Lake JP: The management of complicated diverticulitis and the role of computed tomography. *Am J Gastroenterol.* 2005;100(4):910–7. 10.1111/j.1572-0241.2005.41154.x 15784040

[7] Kruis W Germer CT Leifeld L: Diverticular disease: guidelines of the german society for gastroenterology, digestive and metabolic diseases and the german society for general and visceral surgery. *Digestion.* 2014;90(3):190–207. 10.1159/000367625 25413249

[8] Andeweg CS Mulder IM Felt-Bersma RJ: Guidelines of diagnostics and treatment of acute left-sided colonic diverticulitis. *Dig Surg.* 2013;30(4–6):278–92. 10.1159/000354035 23969324

[9] Cuomo R Barbara G Pace F: Italian consensus conference for colonic diverticulosis and diverticular disease. *United European Gastroenterol J.* 2014;2(5):413–42. 10.1177/2050640614547068 25360320 PMC4212498

[10] Binda GA Cuomo R Laghi A: Practice parameters for the treatment of colonic diverticular disease: Italian Society of Colon and Rectal Surgery (SICCR) guidelines. *Tech Coloproctol.* 2015;19(10):615–26. 10.1007/s10151-015-1370-x 26377584

[11] Wheat CL Strate LL: Trends in Hospitalization for Diverticulitis and Diverticular Bleeding in the United States From 2000 to 2010. *Clin Gastroenterol Hepatol.* 2016;14(1):96–103.e1. 10.1016/j.cgh.2015.03.030 25862988 PMC4624035

[12] Peery AF Dellon ES Lund J: Burden of gastrointestinal disease in the United States: 2012 update. *Gastroenterology.* 2012;143(5):1179–87.e1-3. 10.1053/j.gastro.2012.08.002 22885331 PMC3480553

[13] Bollom A Austrie J Hirsch W: Emergency Department Burden of Diverticulitis in the USA, 2006-2013. *Dig Dis Sci.* 2017;62(10):2694–703. 10.1007/s10620-017-4525-y 28332105 PMC5610055

[14] Mennini FS Sciattella P Marcellusi A: Economic burden of diverticular disease: An observational analysis based on real world data from an Italian region. *Dig Liver Dis.* 2017;49(9):1003–8. 10.1016/j.dld.2017.05.024 28663067

[15] Paterson HM Arnott ID Nicholls RJ: Diverticular disease in Scotland: 2000-2010. *Colorectal Dis.* 2015;17(4):329–34. 10.1111/codi.12811 25359603

[16] van Dijk ST Rottier SJ van Geloven AAW: Conservative Treatment of Acute Colonic Diverticulitis. *Curr Infect Dis Rep.* 2017;19(11):44. 10.1007/s11908-017-0600-y 28942590 PMC5610668

[17] Joliat G Emery J Demartines N: Antibiotic treatment for uncomplicated and mild complicated diverticulitis: outpatient treatment for everyone. *Int J Colorectal Dis.* 2017;32(9):1313–9. 10.1007/s00384-017-2847-z 28664347

[18] Galetin T Galetin A Vestweber KH: Systematic review and comparison of national and international guidelines on diverticular disease. *Int J Colorectal Dis.* 2018;33(3):261–72. 10.1007/s00384-017-2960-z 29349481

[19] Daniels L Ünlü Ç de Korte N: Randomized clinical trial of observational *versus* antibiotic treatment for a first episode of CT-proven uncomplicated acute diverticulitis. *Br J Surg.* 2017;104(1):52–61. 10.1002/bjs.10309 27686365

[20] El-Sayed C Radley S Mytton J: Risk of Recurrent Disease and Surgery Following an Admission for Acute Diverticulitis. *Dis Colon Rectum.* 2018;61(3):382–9. 10.1097/DCR.0000000000000939 29420430

[21] Buchs NC Mortensen NJ Ris F: Natural history of uncomplicated sigmoid diverticulitis. *World J Gastrointest Surg.* 2015;7(11):313–8. 10.4240/wjgs.v7.i11.313 26649154 PMC4663385

[22] Chabok A Andreasson K Nikberg M: Low risk of complications in patients with first-time acute uncomplicated diverticulitis. *Int J Colorectal Dis.* 2017;32(12):1699–702. 10.1007/s00384-017-2912-7 29038965 PMC5691119

[23] Mizrahi I Al-Kurd A Chapchay K: Long-term outcomes of sigmoid diverticulitis: a single-center experience. *J Surg Res.* 2018;221:8–14. 10.1016/j.jss.2017.07.028 29229157

[24] Cohen E Fuller G Bolus R: Increased risk for irritable bowel syndrome after acute diverticulitis. *Clin Gastroenterol Hepatol.* 2013;11(12):1614–9. 10.1016/j.cgh.2013.03.007 23524129 PMC5731449

[25] Lahat A Necula D Yavzori M: Prolonged Recurrent Abdominal Pain is Associated With Ongoing Underlying Mucosal Inflammation in Patients who had an Episode of Acute Complicated Diverticulitis. *J Clin Gastroenterol.* 2018. 10.1097/MCG.0000000000000980 29356787

[26] Andeweg CS Berg R Staal JB: Patient-reported Outcomes After Conservative or Surgical Management of Recurrent and Chronic Complaints of Diverticulitis: Systematic Review and Meta-analysis. *Clin Gastroenterol Hepatol.* 2016;14(2):183–90. 10.1016/j.cgh.2015.08.020 26305068

[27] Peery AF: Colonic Diverticula and Diverticular Disease: 10 Facts Clinicians Should Know. *N C Med J.* 2016;77(3):220–2. 10.18043/ncm.77.3.220 27154895 PMC4994887

[28] Jamal Talabani A Lydersen S Ness-Jensen E: Risk factors of admission for acute colonic diverticulitis in a population-based cohort study: The North Trondelag Health Study, Norway. *World J Gastroenterol.* 2016;22(48):10663–72. 10.3748/wjg.v22.i48.10663 28082819 PMC5192278

[29] Aune D Sen A Leitzmann MF: Body mass index and physical activity and the risk of diverticular disease: a systematic review and meta-analysis of prospective studies. *Eur J Nutr.* 2017;56(8):2423–38. 10.1007/s00394-017-1443-x 28393286 PMC5682875

[30] Strate LL Liu YL Aldoori WH: Obesity increases the risks of diverticulitis and diverticular bleeding. *Gastroenterology.* 2009;136(1):115–122.e1. 10.1053/j.gastro.2008.09.025 18996378 PMC2643271

[31] Humes DJ Ludvigsson JF Jarvholm B: Smoking and the Risk of Hospitalization for Symptomatic Diverticular Disease: A Population-Based Cohort Study from Sweden. *Dis Colon Rectum.* 2016;59(2):110–4. 10.1097/DCR.0000000000000515 26734968

[32] Aune D Sen A Leitzmann MF: Tobacco smoking and the risk of diverticular disease - a systematic review and meta-analysis of prospective studies. *Colorectal Dis.* 2017;19(7):621–33. 10.1111/codi.13748 28556447

[33] Diamant MJ Schaffer S Coward S: Smoking Is Associated with an Increased Risk for Surgery in Diverticulitis: A Case Control Study. *PLoS One.* 2016;11(7):e0153871. 10.1371/journal.pone.0153871 27467077 PMC4965109

[34] Cao Y Strate LL Keeley BR: Meat intake and risk of diverticulitis among men. *Gut.* 2018;67(3):466–72. 10.1136/gutjnl-2016-313082 28069830 PMC5533623

[35] Kvasnovsky CL Papagrigoriadis S Bjarnason I: Increased diverticular complications with nonsteriodal anti-inflammatory drugs and other medications: a systematic review and meta-analysis. *Colorectal Dis.* 2014;16(6):O189–96. 10.1111/codi.12516 24320820

[36] Strate LL Liu YL Huang ES: Use of aspirin or nonsteroidal anti-inflammatory drugs increases risk for diverticulitis and diverticular bleeding. *Gastroenterology.* 2011;140(5):1427–33. 10.1053/j.gastro.2011.02.004 21320500 PMC3081980

[37] Humes DJ Fleming KM Spiller RC: Concurrent drug use and the risk of perforated colonic diverticular disease: a population-based case-control study. *Gut.* 2011;60(2):219–24. 10.1136/gut.2010.217281 20940283

[38] Strate LL Liu YL Aldoori WH: Physical activity decreases diverticular complications. *Am J Gastroenterol.* 2009;104(5):1221–30. 10.1038/ajg.2009.121 19367267 PMC3144158

[39] Crowe FL Balkwill A Cairns BJ: Source of dietary fibre and diverticular disease incidence: a prospective study of UK women. *Gut.* 2014;63(9):1450–6. 10.1136/gutjnl-2013-304644 24385599 PMC4145436

[40] Strate LL: Lifestyle factors and the course of diverticular disease. *Dig Dis.* 2012;30(1):35–45. 10.1159/000335707 22572683

[41] Sköldberg F Svensson T Olén O: A population-based case-control study on statin exposure and risk of acute diverticular disease. *Scand J Gastroenterol.* 2016;51(2):203–10. 10.3109/00365521.2015.1081274 26357870

[42] Maguire LH Song M Strate LE: Higher serum levels of vitamin D are associated with a reduced risk of diverticulitis. *Clin Gastroenterol Hepatol.* 2013;11(12):1631–5. 10.1016/j.cgh.2013.07.035 23954650 PMC3840074

[43] Tursi A Elisei W Picchio M: Serum levels of vitamin D are associated with the severity of the endoscopic appearance of diverticular disease of the colon according to DICA classification. *J Gastrointestin Liver Dis.* 2016;25(4):567–8. 10.15403/jgld.2014.1121.254.vid 27981318

[44] Aldoori WH Giovannucci EL Rimm EB: A prospective study of alcohol, smoking, caffeine, and the risk of symptomatic diverticular disease in men. *Ann Epidemiol.* 1995;5(3):221–8. 10.1016/1047-2797(94)00109-7 7606311

[45] Tønnesen H Engholm G Moller H: Association between alcoholism and diverticulitis. *Br J Surg.* 1999;86(8):1067–8. 10460645 10.1046/j.1365-2168.1999.01171.x

[46] Crowe FL Appleby PN Allen NE: Diet and risk of diverticular disease in Oxford cohort of European Prospective Investigation into Cancer and Nutrition (EPIC): prospective study of British vegetarians and non-vegetarians. *BMJ.* 2011;343:d4131. 10.1136/bmj.d4131 21771850 PMC3139912

[47] Morris CR Harvey IM Stebbings WS: Do calcium channel blockers and antimuscarinics protect against perforated colonic diverticular disease? A case control study. *Gut.* 2003;52(12):1734–7. 10.1136/gut.52.12.1734 14633952 PMC1773902

[48] Nguyen GC Sam J Anand N: Epidemiological trends and geographic variation in hospital admissions for diverticulitis in the United States. *World J Gastroenterol.* 2011;17(12):1600–5. 10.3748/wjg.v17.i12.1600 21472127 PMC3070132

[49] Chang SS Huang N Hu HY: Patients with end-stage renal disease were at an increased risk of hospitalization for acute diverticulitis. *Medicine (Baltimore).* 2016;95(39):e4881. 10.1097/MD.0000000000004881 27684821 PMC5265914

[50] Nikberg M Ji J Leppert J: Socioeconomic characteristics and comorbidities of diverticular disease in Sweden 1997-2012. *Int J Colorectal Dis.* 2017;32(11):1591–6. 10.1007/s00384-017-2853-1 28785818 PMC5635093

[51] Strate LL Erichsen R Baron JA: Heritability and familial aggregation of diverticular disease: a population-based study of twins and siblings. *Gastroenterology.* 2013;144(4):736–742.e1; quiz e14. 10.1053/j.gastro.2012.12.030 23313967

[52] Connelly TM Berg AS Hegarty JP: The TNFSF15 gene single nucleotide polymorphism rs7848647 is associated with surgical diverticulitis. *Ann Surg.* 2014;259(6):1132–7. 10.1097/SLA.0000000000000232 24814505

[53] Coble JL Sheldon KE Yue F: Identification of a rare *LAMB4* variant associated with familial diverticulitis through exome sequencing. *Hum Mol Genet.* 2017;26(16):3212–20. 10.1093/hmg/ddx204 28595269 PMC6251658

[54] Strate LL Liu YL Syngal S: Nut, corn, and popcorn consumption and the incidence of diverticular disease. *JAMA.* 2008;300(8):907–14. 10.1001/jama.300.8.907 18728264 PMC2643269

[55] Carabotti M Cuomo R Barbara G: Demographic and clinical features distinguish subgroups of diverticular disease patients: Results from an Italian nationwide registry. *United European Gastroenterol J.* 2018;43:205064061876495 10.1177/2050640618764953 PMC6047280 30023071

[56] Hupfeld L Burcharth J Pommergaard HC: Risk factors for recurrence after acute colonic diverticulitis: a systematic review. *Int J Colorectal Dis.* 2017;32(5):611–22. 10.1007/s00384-017-2766-z 28110383

[57] Wedel T Barrenschee M Lange C: Morphologic Basis for Developing Diverticular Disease, Diverticulitis, and Diverticular Bleeding. *Viszeralmedizin.* 2015;31(2):76–82. 10.1159/000381431 26989376 PMC4789973

[58] Walker MM: Inflammation, Genetics, Dysbiosis, and the Environment: New Paradigms for Diagnosis in Complex Chronic Gut Syndromes. *J Clin Gastroenterol.* 2016;50 Suppl 1:S4–5. 10.1097/MCG.0000000000000613 27622361

[59] Rezapour M Ali S Stollman N: Diverticular Disease: An Update on Pathogenesis and Management. *Gut Liver.* 2018;12(2):125–32. 10.5009/gnl16552 28494576 PMC5832336

[60] Daniels L Philipszoon LE Boermeester MA: A hypothesis: important role for gut microbiota in the etiopathogenesis of diverticular disease. *Dis Colon Rectum.* 2014;57(4):539–43. 10.1097/DCR.0000000000000078 24608313

[61] Barbara G Scaioli E Barbaro MR: Gut microbiota, metabolome and immune signatures in patients with uncomplicated diverticular disease. *Gut.* 2017;66(7):1252–61. 10.1136/gutjnl-2016-312377 27618836

[62] Thevaranjan N Puchta A Schulz C: Age-Associated Microbial Dysbiosis Promotes Intestinal Permeability, Systemic Inflammation, and Macrophage Dysfunction. *Cell Host Microbe.* 2017;21:455–466.e4. 10.1016/j.chom.2017.03.002 28407483 PMC5392495

[63] Koeth RA Wang Z Levison BS: Intestinal microbiota metabolism of *L*-carnitine, a nutrient in red meat, promotes atherosclerosis. *Nat Med.* 2013;19(5):576–85. 10.1038/nm.3145 23563705 PMC3650111

[64] Le Leu RK Young GP Hu Y: Dietary red meat aggravates dextran sulfate sodium-induced colitis in mice whereas resistant starch attenuates inflammation. *Dig Dis Sci.* 2013;58(12):3475–82. 10.1007/s10620-013-2844-1 23990000

[65] Breton J Plé C Guerin-Deremaux L: Intrinsic immunomodulatory effects of low-digestible carbohydrates selectively extend their anti-inflammatory prebiotic potentials. *Biomed Res Int.* 2015;2015:162398. 10.1155/2015/162398 25977916 PMC4419225

[66] David LA Maurice CF Carmody RN: Diet rapidly and reproducibly alters the human gut microbiome. *Nature.* 2014;505(7484):559–63. 10.1038/nature12820 24336217 PMC3957428

[67] Lamps LW Knapple WL: Diverticular disease-associated segmental colitis. *Clin Gastroenterol Hepatol.* 2007;5(1):27–31. 10.1016/j.cgh.2006.10.024 17234553

[68] von Rahden BH Kircher S Thiery S: Association of steroid use with complicated sigmoid diverticulitis: potential role of activated CD68+/CD163+ macrophages. *Langenbecks Arch Surg.* 2011;396(6):759–68. 10.1007/s00423-011-0797-4 21553154

[69] Turco F Andreozzi P Palumbo I: Bacterial stimuli activate nitric oxide colonic mucosal production in diverticular disease. Protective effects of *L. casei DG*® ( *Lactobacillus paracasei* CNCM I-1572). *United European Gastroenterol J.* 2017;5(5):715–24. 10.1177/2050640616684398 28815036 PMC5548353

[70] Peery AF Keku TO Addamo C: Colonic Diverticula Are Not Associated With Mucosal Inflammation or Chronic Gastrointestinal Symptoms. *Clin Gastroenterol Hepatol.* 2018;16(6):884–891.e1. 10.1016/j.cgh.2017.05.051 28603053 PMC5722710

[71] Zeng MY Inohara N Nuñez G: Mechanisms of inflammation-driven bacterial dysbiosis in the gut. *Mucosal Immunol.* 2017;10(1):18–26. 10.1038/mi.2016.75 27554295 PMC5788567

[72] Guarino MP Cicala M Putignani L: Gastrointestinal neuromuscular apparatus: An underestimated target of gut microbiota. *World J Gastroenterol.* 2016;22(45):9871–9. 10.3748/wjg.v22.i45.9871 28018095 PMC5143755

[73] Bassotti G Villanacci V: Colonic diverticular disease: abnormalities of neuromuscular function. *Dig Dis.* 2012;30(1):24–8. 10.1159/000335702 22572681

[74] Bassotti G Battaglia E Spinozzi F: Twenty-four hour recordings of colonic motility in patients with diverticular disease: evidence for abnormal motility and propulsive activity. *Dis Colon Rectum.* 2001;44(12):1814–20. 10.1007/BF02234460 11742167

[75] Fornai M Colucci R Antonioli L: Role of cyclooxygenase isoforms in the altered excitatory motor pathways of human colon with diverticular disease. *Br J Pharmacol.* 2014;171(15):3728–40. 10.1111/bph.12733 24758697 PMC4128069

[76] Simpson J Sundler F Humes DJ: Post inflammatory damage to the enteric nervous system in diverticular disease and its relationship to symptoms. *Neurogastroenterol Motil.* 2009;21(8):847–e58. 10.1111/j.1365-2982.2009.01308.x 19453515

[77] Golder M Burleigh DE Belai A: Smooth muscle cholinergic denervation hypersensitivity in diverticular disease. *Lancet.* 2003;361(9373):1945–51. 10.1016/S0140-6736(03)13583-0 12801738

[78] Golder M Burleigh DE Ghali L: Longitudinal muscle shows abnormal relaxation responses to nitric oxide and contains altered levels of NOS1 and elastin in uncomplicated diverticular disease. *Colorectal Dis.* 2007;9(3):218–28. 10.1111/j.1463-1318.2006.01160.x 17298619

[79] Whiteway J Morson BC: Elastosis in diverticular disease of the sigmoid colon. *Gut.* 1985;26(3):258–66. 10.1136/gut.26.3.258 3972272 PMC1432643

[80] Hellwig I Böttner M Barrenschee M: Alterations of the enteric smooth musculature in diverticular disease. *J Gastroenterol.* 2014;49(8):1241–52. 10.1007/s00535-013-0886-y 24113817

[81] Deduchovas O Saladzinskas Z Tamelis A: Morphologic pattern of myenteric neural plexus in colonic diverticular disease. A whole-mount study employing histochemical staining for acetylcholinesterase. *Ann Anat.* 2008;190(6):525–30. 10.1016/j.aanat.2008.09.002 19026527

[82] Iwase H Sadahiro S Mukoyama S: Morphology of myenteric plexuses in the human large intestine: comparison between large intestines with and without colonic diverticula. *J Clin Gastroenterol.* 2005;39(8):674–8. 10.1097/01.mcg.0000173856.84814.37 16082275

[83] Wedel T Büsing V Heinrichs G: Diverticular disease is associated with an enteric neuropathy as revealed by morphometric analysis. * Neurogastroenterol Motil.* 2010;22(4):407–14, e93-4. 10.1111/j.1365-2982.2009.01445.x 20040058

[84] Pantaroto M Lopes Filho Gde J Pinto CA: Comparative study of collagen deposition in the colon wall of patients operated for sigmoid diverticular disease. *Acta Cir Bras.* 2015;30(10):715–9. 10.1590/S0102-865020150100000010 26560431

[85] Pallotta L Scirocco A Ignazzi A: Inflammatory and phenotypic smooth muscle alterations in colonic diverticulosis and diverticular disease. [abstract] *Gastroenterology.* 2017;152(5, Supplement 1):S50 10.1016/S0016-5085(17)30527-9

[86] Brown SR Cleveland EM Deeken CR: Type I/type III collagen ratio associated with diverticulitis of the colon in young patients. *J Surg Res.* 2017;207:229–34. 10.1016/j.jss.2016.08.044 27979482

[87] Galligan JJ Sternini C: Insights into the Role of Opioid Receptors in the GI Tract: Experimental Evidence and Therapeutic Relevance. *Handb Exp Pharmacol.* 2017;239:363–78. 10.1007/164_2016_116 28204957 PMC6310692

[88] Martinez-Cutillas M Mañé N Gallego D: EP _2_ and EP _4_ receptors mediate PGE _2_ induced relaxation in murine colonic circular muscle: pharmacological characterization. *Pharmacol Res.* 2014;90:76–86. 10.1016/j.phrs.2014.10.001 25461458

[89] Gummesson A Carlsson LM Storlien LH: Intestinal permeability is associated with visceral adiposity in healthy women. *Obesity (Silver Spring).* 2011;19(11):2280–2. 10.1038/oby.2011.251 21852815

[90] Büning C von Kraft C Hermsdorf M: Visceral Adipose Tissue in Patients with Crohn's Disease Correlates with Disease Activity, Inflammatory Markers, and Outcome. *Inflamm Bowel Dis.* 2015;21(11):2590–7. 10.1097/MIB.0000000000000527 26222339

[91] Paeschke A Erben U Kredel LI: Role of visceral fat in colonic inflammation: from Crohn's disease to diverticulitis. *Curr Opin Gastroenterol.* 2017;33(1):53–8. 10.1097/MOG.0000000000000324 27798440

[92] Docimo S Jr Lee Y Chatani P: Visceral to subcutaneous fat ratio predicts acuity of diverticulitis. *Surg Endosc.* 2017;31(7):2808–12. 10.1007/s00464-016-5290-2 27778168

[93] Tattoli I Petitta C Scirocco A: Microbiota, innate immune system, and gastrointestinal muscle: ongoing studies. *J Clin Gastroenterol.* 2012;46 Suppl:S6–11. 10.1097/MCG.0b013e318265ea7d 22955360

[94] Matarrese P Petitta C Scirocco A: Antioxidants counteract lipopolysaccharide-triggered alterations of human colonic smooth muscle cells. *Free Radic Biol Med.* 2012;53(11):2102–11. 10.1016/j.freeradbiomed.2012.09.022 23044262

[95] Scirocco A Matarrese P Carabotti M: Cellular and Molecular Mechanisms of Phenotypic Switch in Gastrointestinal Smooth Muscle. *J Cell Physiol.* 2016;231(2):295–302. 10.1002/jcp.25105 26206426

[96] Böttner M Wedel T: Abnormalities of neuromuscular anatomy in diverticular disease. *Dig Dis.* 2012;30(1):19–23. 10.1159/000335699 22572680

[97] Gordon IO Agrawal N Willis E: Fibrosis in ulcerative colitis is directly linked to severity and chronicity of mucosal inflammation. *Aliment Pharmacol Ther.* 2018;47(7):922–39. 10.1111/apt.14526 29411405 PMC5842117

[98] Dickerson EC Chong ST Ellis JH: Recurrence of Colonic Diverticulitis: Identifying Predictive CT Findings-Retrospective Cohort Study. *Radiology.* 2017;285(3):850–8. 10.1148/radiol.2017161374 28837412

[99] Frye JM Hansel SL Dolan SG: NSAID enteropathy: appearance at CT and MR enterography in the age of multi-modality imaging and treatment. *Abdom Imaging.* 2015;40(5):1011–25. 10.1007/s00261-015-0367-2 25666969

[100] Bjarnason I Scarpignato C Holmgren E: Mechanisms of Damage to the Gastrointestinal Tract From Nonsteroidal Anti-Inflammatory Drugs. *Gastroenterology.* 2018;154(3):500–14. 10.1053/j.gastro.2017.10.049 29221664

[101] Price JC Tien PC: *Editorial*: Statins and Liver Disease: Is it Time to Recommend Statins to Prevent Liver Disease Progression? *Am J Gastroenterol.* 2017;112(10):1506–7. 10.1038/ajg.2017.250 28978973 PMC8842818

[102] Li D de Mestral C Baxter NN: Risk of readmission and emergency surgery following nonoperative management of colonic diverticulitis: a population-based analysis. *Ann Surg.* 2014;260(3):423–30; discussion 430-1. 10.1097/SLA.0000000000000870 25115418

[103] van Randen A Laméris W van Es HW: A comparison of the accuracy of ultrasound and computed tomography in common diagnoses causing acute abdominal pain. *Eur Radiol.* 2011;21(7):1535–45. 10.1007/s00330-011-2087-5 21365197 PMC3101356

[104] Chabok A Påhlman L Hjern F: Randomized clinical trial of antibiotics in acute uncomplicated diverticulitis. *Br J Surg.* 2012;99(4):532–9. 10.1002/bjs.8688 22290281

[105] van Dijk ST Daniels L Ünlü Ç: Long-Term Effects of Omitting Antibiotics in Uncomplicated Acute Diverticulitis. *Am J Gastroenterol.* 2018. 10.1038/s41395-018-0030-y 29700480

[106] Andersen JC Bundgaard L Elbrønd H: Danish national guidelines for treatment of diverticular disease. *Dan Med J.* 2012;59(5):C4453. 22549495

[107] Pietrzak A Bartnik W Szczepkowski M: Polish interdisciplinary consensus on diagnostics and treatment of colonic diverticulosis (2015). *Pol Przegl Chir.* 2015;87(4):203–20. 10.1515/pjs-2015-0045 26146121

[108] Stollman N Smalley W Hirano I: American Gastroenterological Association Institute Guideline on the Management of Acute Diverticulitis. *Gastroenterology.* 2015;149(7):1944–9. 10.1053/j.gastro.2015.10.003 26453777

[109] Sánchez-Velázquez P Grande L Pera M: Outpatient treatment of uncomplicated diverticulitis: a systematic review. *Eur J Gastroenterol Hepatol.* 2016;28(6):622–7. 10.1097/MEG.0000000000000610 26891198

[110] van de Wall BJM Stam MAW Draaisma WA: Surgery versus conservative management for recurrent and ongoing left-sided diverticulitis (DIRECT trial): an open-label, multicentre, randomised controlled trial. *Lancet Gastroenterol Hepatol.* 2017;2(1):13–22. 10.1016/S2468-1253(16)30109-1 28404008

[111] Morris AM Regenbogen SE Hardiman KM: Sigmoid diverticulitis: a systematic review. *JAMA.* 2014;311(3):287–97. 10.1001/jama.2013.282025 24430321

[112] Pfützer RH Kruis W: Management of diverticular disease. *Nat Rev Gastroenterol Hepatol.* 2015;12(11):629–38. 10.1038/nrgastro.2015.115 26170219

[113] Jeger V Pop R Forudastan F: Is there a role for procalcitonin in differentiating uncomplicated and complicated diverticulitis in order to reduce antibiotic therapy? A prospective diagnostic cohort study. *Swiss Med Wkly.* 2017;147:w14555. 10.4414/smw.2017.14555 29185246

[114] Reynolds IS Heaney RM Khan W: The Utility of Neutrophil to Lymphocyte Ratio as a Predictor of Intervention in Acute Diverticulitis. *Dig Surg.* 2017;34(3):227–32. 10.1159/000450836 27941316

[115] Buchs NC Konrad-Mugnier B Jannot AS: Assessment of recurrence and complications following uncomplicated diverticulitis. *Br J Surg.* 2013;100(7):976–9; discussion 979. 10.1002/bjs.9119 23592303

[116] Jaung R Kularatna M Robertson JP: Uncomplicated Acute Diverticulitis: Identifying Risk Factors for Severe Outcomes. *World J Surg.* 2017;41(9):2258–65. 10.1007/s00268-017-4012-9 28401253

[117] Carabotti M Annibale B: Treatment of diverticular disease: an update on latest evidence and clinical implications. *Drugs Context.* 2018;7:212526. 10.7573/dic.212526 29623099 PMC5866096

[118] Banasiewicz T Francuzik W Bobkiewicz A: The influence of rifaximin on diverticulitis rate and quality of life in patients with diverticulosis. *Pol Przegl Chir.* 2017;89(1):22–31. 10.5604/01.3001.0009.6012 28522790

[119] Maconi G Barbara G Bosetti C: Treatment of diverticular disease of the colon and prevention of acute diverticulitis: a systematic review. *Dis Colon Rectum.* 2011;54(10):1326–38. 10.1097/DCR.0b013e318223cb2b 21904150

[120] Lanas A Ponce J Bignamini A: One year intermittent rifaximin plus fibre supplementation vs. fibre supplementation alone to prevent diverticulitis recurrence: a proof-of-concept study. *Dig Liver Dis.* 2013;45(2):104–9. 10.1016/j.dld.2012.09.006 23092785

[121] Tursi A Brandimarte G Elisei W: Randomised clinical trial: mesalazine and/or probiotics in maintaining remission of symptomatic uncomplicated diverticular disease--a double-blind, randomised, placebo-controlled study. *Aliment Pharmacol Ther.* 2013;38(7):741–51. 10.1111/apt.12463 23957734

[122] Raskin JB Kamm MA Jamal MM: Mesalamine did not prevent recurrent diverticulitis in phase 3 controlled trials. *Gastroenterology.* 2014;147(4):793–802. 10.1053/j.gastro.2014.07.004 25038431

[123] Kruis W Kardalinos V Eisenbach T: Randomised clinical trial: mesalazine versus placebo in the prevention of diverticulitis recurrence. *Aliment Pharmacol Ther.* 2017;46(3):282–91. 10.1111/apt.14152 28543263 PMC5518301

[124] Carter F Alsayb M Marshall JK: Mesalamine (5-ASA) for the prevention of recurrent diverticulitis. *Cochrane Database Syst Rev.* 2017;10:CD009839. 10.1002/14651858.CD009839.pub2 28973845 PMC6485423

[125] Khan MA Ali B Lee WM: Mesalamine Does Not Help Prevent Recurrent Acute Colonic Diverticulitis: Meta-Analysis of Randomized, Placebo-Controlled Trials. *Am J Gastroenterol.* 2016;111(4):579–81. 10.1038/ajg.2016.24 27125717

[126] Uno Y van Velkinburgh JC: Logical hypothesis: Low FODMAP diet to prevent diverticulitis. *World J Gastrointest Pharmacol Ther.* 2016;7(4):503–12. 10.4292/wjgpt.v7.i4.503 27867683 PMC5095569

